# What are the main inefficiencies in trial conduct? A survey of staff at registered clinical trials units

**DOI:** 10.1186/1745-6215-16-S2-P181

**Published:** 2015-11-16

**Authors:** Lelia Duley, Alison McDonald, Alexa Gillman

**Affiliations:** 1 Nottingham Clinical Trials Unit, University of Nottingham, Nottingham, UK; 2 Centre for Healthcare Randomised Trials (CHaRT), University of Aberdeen, Aberdeen, UK; 3 The Institute of Cancer Research CTSU, London, UK

## Background

The Efficient Trial Conduct subgroup of the Work Programme of the UKCRC Registered Clinical Trials Units (CTUs) Network aimed to identify inefficiencies during the two key stages of the trial conduct life cycle: (i) from grant award to first participant, (ii) from first participant to final reporting of results.

## Methods

An online survey we sent to email lists for Quality Assurance, Information Systems, statistics, trial managers, and pharmacovigilance staff at registered clinical trials units. An email reminder was sent after two weeks.

## Results

There were 43 respondents from 25 registered CTUs; one third were trial managers. From grant award to first participant R&D approvals were reported as a top inefficiency by 23 respondents, contracts by 22 and other approvals by 13. Site selection, feasibility, piloting at site, and site training were also issues.

From recruitment of first patient to publication the top inefficiency was recruitment targets not met, with data collection (including CRF design) the next most common, followed by writing up. Delays in approvals for new sites and poor planning were also issues.

## Discussion

Efficiency of trial conduct would be improved further improvement in the approvals process, better training of site staff, improved working relationships between Chief Investigators and CTUs, and by sharing good practice across CTUs. Better information is needed about how to improve the efficiency and quality of trial conduct.

